# Identification of a Feed-Forward Loop Between 15(S)-HETE and PGE2 in Human Amnion at Parturition

**DOI:** 10.1016/j.jlr.2022.100294

**Published:** 2022-10-04

**Authors:** Fan Zhang, Kang Sun, Wang-Sheng Wang

**Affiliations:** 1 Center for Reproductive Medicine, Ren Ji Hospital, School of Medicine, Shanghai Jiao Tong University, Shanghai, P.R.China; 2 Shanghai Key Laboratory for Assisted Reproduction and Reproductive Genetics, Shanghai, P.R.China

**Keywords:** 15(S)-hydroxyeicosatetraenoic acid, lipoxygenase 15, cyclooxygenase-2, arachidonic acid, inflammation, eicosanoids, metabolomics, lipopolysaccharide, interleukin-1β, serum amyloid A1, AA, arachidonic acid, ALOX, lipoxygenase, COX, cyclooxygenase, dpc, days post-coitus, IL-1β, interleukin-1β, LPS, lipopolysaccharide, mPGES-1, microsomal prostaglandin E synthase-1, PGE2, prostaglandin E2, PG, prostaglandin, PL, preterm labor, PNL, preterm nonlabor, PPARγ, peroxisome proliferator-activated receptor γ, qRT-PCR, quantitative real-time polymerase chain reaction, SAA1, serum amyloid A1, TL, term labor, TNL, term nonlabor

## Abstract

Human parturition is associated with massive arachidonic acid (AA) mobilization in the amnion, indicating that large amounts of AA-derived eicosanoids are required for parturition. Prostaglandin E2 (PGE2) synthesized from the cyclooxygenase (COX) pathway is the best characterized AA-derived eicosanoid in the amnion which plays a pivotal role in parturition. The existence of any other pivotal AA-derived eicosanoids involved in parturition remains elusive. Here, we screened such eicosanoids in human amnion tissue with AA-targeted metabolomics and studied their role and synthesis in parturition by using human amnion fibroblasts and a mouse model. We found that lipoxygenase (ALOX) pathway-derived 15(S)-hydroxyeicosatetraenoic acid (15(S)-HETE) and its synthetic enzymes ALOX15 and ALOX15B were significantly increased in human amnion at parturition. Although 15(S)-HETE is ineffective on its own, it potently potentiated the activation of NF-κB by inflammatory mediators including lipopolysaccharide, interleukin-1β, and serum amyloid A1, resulting in the amplification of COX-2 expression and PGE2 production in amnion fibroblasts. In turn, we determined that PGE2 induced ALOX15/15B expression and 15(S)-HETE production through its EP2 receptor-coupled PKA pathway, thereby forming a feed-forward loop between 15(S)-HETE and PGE2 production in the amnion at parturition. Our studies in pregnant mice showed that 15(S)-HETE injection induced preterm birth with increased COX-2 and PGE2 abundance in the fetal membranes and placenta. Conclusively, 15(S)-HETE is identified as another crucial parturition-pertinent AA-derived eicosanoid in the amnion, which may form a feed-forward loop with PGE2 in parturition. Interruption of this feed-forward loop may be of therapeutic value for the treatment of preterm birth.

## Graphical abstract

**Figure:**
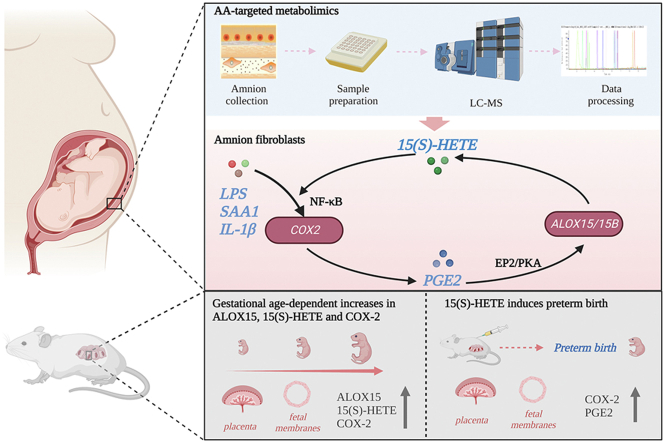

Preterm birth remains to be the leading cause of perinatal mortality and morbidity due to lack of reliable predictive and preventive measures, which attributes largely to the insufficient understanding of the initiating mechanism of human parturition (1). Accumulating evidence indicates that human parturition can be initiated by a myriad of signals originated from multiple maternal and fetal tissues (2, 3, 4). Among them, signals originated from the fetal membranes are worthy of particular attention. The importance of fetal membranes-derived signals in labor onset is very well illustrated by the high incidence of preterm birth in chorioamnionitis, a condition of membrane infection which accounts for approximately one-third of preterm birth (5, 6). Among the signals derived from the fetal membranes, prostaglandins (PGs), particularly prostaglandin E2 (PGE2) formed from arachidonic acid (AA) through the cyclooxygenase (COX) pathway (Fig. 1A) in the amnion layer, are one of the best characterized labor initiating signals (7, 8, 9, 10). PGE2 is known to participate in the initiation of parturition by stimulating myometrial contraction, cervical ripening, and fetal membrane activation (11, 12, 13). Inflammatory mediators are known to be the major cause of increased PGE2 synthesis in parturition (14, 15, 16). Depending on the presence or absence of infection, inflammation of the fetal membranes can either be infectious as in chorioamnionitis or sterile as in normal parturition (6, 17, 18). Irrespective of the nature of inflammation, activation of the proinflammatory transcription factor NF-κB with consequently increased expression of COX-2, the rate-limiting enzyme in PG synthesis, is recognized as the primary mechanism accounting for the upregulation of PGE2 production by inflammatory mediators in the amnion at parturition (19, 20, 21).

**Fig. 1:**
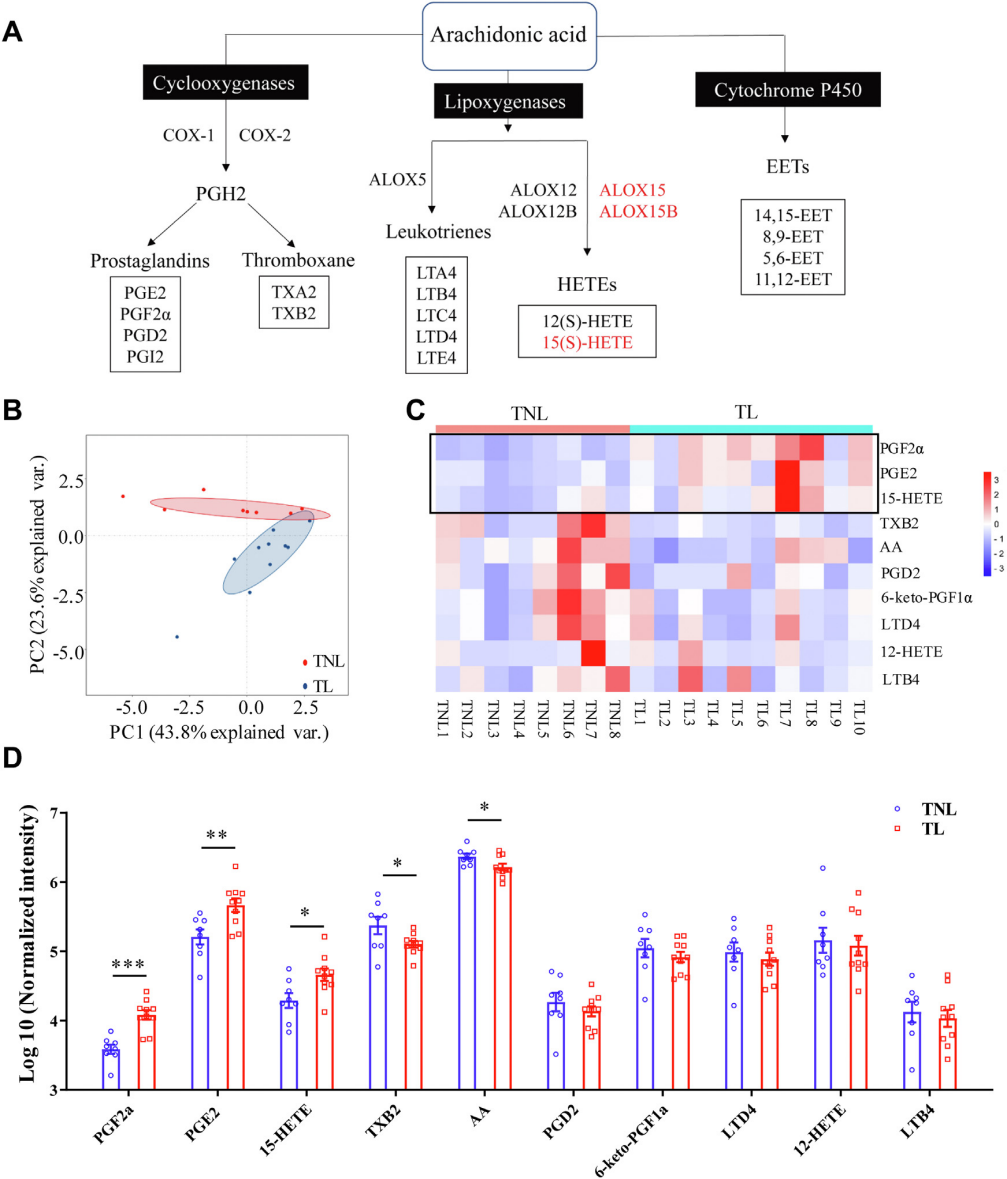
Changes of AA-derived eicosanoids in human amnion in parturition as revealed with AA-targeted metabolomics. A: The diagram depicting the three major pathways of AA metabolism. B: Principal component analysis of the data derived from AA-targeted metabolomics. Principal component (PC) 1 and 2 explain 43.8% and 23.6% of the total variance of the full dataset, respectively. Pink and blue colors code TNL (term delivery without labor) and TL (term delivery with labor) groups, respectively. C: Heat map displaying 10 AA metabolites detected in the amnion obtained from TNL (n = 8) and TL (n = 10). Blue to red represents metabolite level from low to high. Boxed part indicates the three eicosanoids significantly increased in parturition. D: Histogram displaying the abundance of 10 detected AA metabolites in the amnion obtained from TL (n = 10) and TNL (n = 8). Statistical analysis was performed with unpaired Student’s *t*-test. ∗*P*<0.05, ∗∗*P*<0.01, ∗∗∗*P*<0.001 versus TNL. AA, arachidonic acid; TNL, term nonlabor; TL, term labor.

The amnion is particularly rich in AA, which is greatly depleted during parturition for the synthesis of bioactive eicosanoids in the initiation of parturition (22, 23). Although the amnion is known to synthesize the most PGE2 among gestational tissues in parturition (7, 10, 24), a spectrum of other eicosanoids including leukotrienes, HETEs, and epoxyeicosatrienoic acids can also be formed from AA through the lipoxygenase (ALOX) and cytochrome P450 enzyme pathways (Fig. 1A) (25, 26). Yet, very little is known about the role of these AA-derived eicosanoids in parturition. Identification of such eicosanoids may help us gain further insight into the mechanism underlying labor onset in humans, which may offer potential therapeutic targets for preterm birth.

In this study, we performed AA-targeted metabolomic study in human amnion with an aim to screen such crucial eicosanoids involved in human parturition. We identified that 15(S)-HETE, formed from the ALOX pathway under the enzymatic action of ALOX15 and ALOX15B (Fig. 1A) (25, 26, 27, 28), might be another important eicosanoid associated with parturition. Studies in human amnion fibroblasts showed that 15(S)-HETE participated in parturition by potentiating proinflammatory mediators-induced activation of the NF-κB/COX-2/PGE2 pathway, and in turn, PGE2 further drove 15(S)-HETE production through induction of ALOX15/15B expression, leading to the formation of a feed-forward loop between 15(S)-HETE and PGE2 syntheses in the amnion at parturition. The parturition-initiating effect of 15(S)-HETE was further illustrated in pregnant mice.

## Materials and Methods

### Recruitment of pregnant women

This study was performed in accordance with the Declaration of Helsinki. Human fetal membranes and placental villous tissues were obtained from pregnant women with written informed consent under a protocol (No. [2013] N025) approved by the Ethics Committee of Ren Ji Hospital, School of Medicine, Shanghai Jiao Tong University. Participating subjects were classified into four groups: elective cesarean section without labor at term (designated as term nonlabor group, TNL); spontaneous labor at term (designated as term labor group, TL); preterm pregnancies terminated by cesarean section without labor for maternal or fetal conditions including placenta previa, vasa previa, and fetal distress (designated as preterm nonlabor group, PNL); and spontaneous preterm labor with no indication of infection (designated as preterm labor group, PL). Upon deliveries, the fetal membranes either within spontaneous or artificial rupture site and the villous tissue from the maternal side of the placenta were collected immediately. The fetal membranes were further separated into the amnion and chorion/decidua layers. The detailed information of recruited women was listed in supplemental Tables S1–S3.

### AA-targeted metabolomics in human amnion

The amnion within the artificial rupture site from TNL (n = 8) and the spontaneous rupture site from TL (n = 10) was used to screen AA-derived eicosanoids pertinent to human parturition using AA-targeted metabolomics. The amnion tissue was homogenized in precipitating solvent (1:4 water: acetonitrile) followed by sonication on ice for 30 min. After centrifugation, the supernatant was collected for extraction of AA analogs with the Oasis HLB elution system (Waters, Milford, MA) which was preactivated and equilibrated with methanol and water, respectively. After elution with methanol, the eluted AA analogs were lyophilized and reconstituted in 1% formic acid and 80% acetonitrile solution for analysis with high-performance LC-MS on a UPLC I-Class PLUS System (Waters, Milford, MA) coupled with the 5500 QTRAP system (AB SCIEX, Framingham, MA). Quantitative control samples were used to monitor the stability and repeatability of the system. MultiQuant software was used to extract chromatographic peak area and retention time. The relative quantitative analysis of each AA metabolite was based on the peak area.

### Measurement of 15(S)-HETE in human amnion with ELISA

To confirm the data of AA-targeted metabolomics, the amnion within the artificial rupture site (TNL, n = 14; PNL, n = 10) and spontaneous rupture site (TL, n = 14; PL, n = 10) was collected from deliveries with or without labor at term or preterm. The tissue was snap-frozen in liquid nitrogen and extracted with ethyl acetate after homogenization. After evaporation of ethyl acetate, the extract was reconstituted in the assay buffer for measurement of 15(S)-HETE with an ELISA kit (Cayman Chemical, Ann Arbor, MI) according to the manufacturer’s protocol.

### Measurement of ALOX15 and ALOX15B in human amnion with quantitative real-time polymerase chain reaction and Western blotting

To observe whether the abundance of ALOX15 and ALOX15B mRNA and protein changed consistently in the amnion in labor at term and preterm, the amnion within the artificial rupture site (TNL and PNL) and spontaneous rupture site (TL and PL) was collected and snap-frozen in liquid nitrogen for total RNA and protein extraction. The abundance of ALOX15 and ALOX15B protein in the chorion/decidua and placental villous tissues was also compared between TL and TNL groups.

For RNA extraction, the snap-frozen tissue was grounded and homogenized with a total RNA isolation Kit (Foregene, Chengdu, China). After examination of RNA quality, reverse transcription was carried out using a Prime-Script RT Master Mix Kit (TaKaRa, Kyoto, Japan). The amount of *ALOX15* and *ALOX15B* mRNA was determined with quantitative real-time polymerase chain reaction (qRT-PCR) using the above reverse-transcribed cDNA and power SYBR® Premix Ex Taq™ (TaKaRa). Housekeeping gene *GAPDH* was amplified in parallel as an internal control. The relative mRNA abundance was quantitated using the 2-^△△Ct^ method. The primer sequences used for qRT-PCR are illustrated in supplemental Table S4.

For protein extraction, the snap-frozen tissue was grounded and homogenized in ice-cold RIPA lysis buffer (Active Motif, Carlsbad, CA) containing inhibitors for protease (Roche, Indianapolis, IN) and phosphatase (Roche). After centrifugation, the supernatant was collected for determination of protein concentration using the Bradford method. The abundance of ALOX15 and AOX15B protein was determined with Western blotting. Briefly, 30 μg protein from each sample was electrophoresed in a sodium dodecyl sulfate-polyacrylamide gel. After transferring to a nitrocellulose membrane blot, the blot was blocked with 5% nonfat milk and incubated with antibodies against ALOX15 (1:500; Thermo Fisher, Waltham, MA; #MA5-25853) and ALOX15B (1:500; Thermo Fisher; #PA5-97456) overnight at 4°C, followed by incubation with appropriate horseradish peroxidase-conjugated secondary antibodies. Peroxidase activity was developed with a chemiluminescence detection system (Millipore, Billerica, MA) and visualized using a G-Box chemiluminescence image capture system (Syngene, Cambridge, UK). Internal loading control was performed by probing the same blot with an antibody against the housekeeping protein GAPDH (1:10,000; Proteintech, Wuhan, China; #60004-1). The ratio of band intensities of ALOX15 and ALOX15B to that of GAPDH was used to indicate ALOX15 and ALOX15B protein abundance.

### Immunohistochemical staining of ALOX15 and ALOX15B in human amnion tissue

Immunohistochemical staining was carried out on paraffin-embedded amnion tissue sections prepared from TNL (n = 3). After deparaffinization and quenching the endogenous peroxidase activity with 0.3% H_2_O_2_, the section was incubated with normal serum to block the nonspecific binding site, followed by incubation with primary antibodies against human ALOX15 (Thermo Fisher; #MA5-25853) and ALOX15B (Thermo Fisher; #PA5-97456) at 1:100 dilution or with nonimmune serum (Proteintech) for negative control overnight at 4°C. After washing, the section was incubated consecutively with a corresponding biotinylated secondary antibody and the avidin-biotin complex conjugated with horseradish peroxidase (Vector Laboratories, Burlingame, CA). The horseradish peroxidase activity was developed as a red color with the substrate 3-amino-9-ethyl carbazole (Vector Laboratories). The slide was counterstained with hematoxylin (blue color) and examined under a regular bright field microscope (Zeiss, Oberkochen, Germany).

### Isolation and culture of human amnion fibroblasts and epithelial cells

The entire amnion from the reflected membranes obtained from TNL was used for isolation of amnion fibroblasts and epithelial cells. Briefly, the amnion tissue was digested twice with 0.125% trypsin (Life Technologies Inc, Grand Island, NY) and then washed vigorously with normal saline for isolation of epithelial cells. The epithelial cells in trypsin-digested medium and normal saline wash were collected by centrifugation. The remaining amnion mesenchymal tissue was further digested with 0.1% collagenase (Sigma, St. Louis, MO) to isolate fibroblasts. The isolated amnion epithelial cells and fibroblasts were resuspended and cultured in DMEM containing 10% FBS and antibiotics (Life Technologies Inc). This method of amnion cell isolation yields high purity of epithelial cells (> 99%) and fibroblasts (> 95%), which have been previously characterized by staining of cytokeratin-7, vimentin, and CD45, markers for epithelial, mesenchymal, and immune cells, respectively (29).

### Treatment of human amnion cells

To compare the abundance of ALOX15 and ALOX15B between amnion fibroblasts and epithelial cells, the cells were cultured for 3 days and then subjected to protein extraction for analysis with Western blotting using antibodies against ALOX15 (1:500) and ALOX15B (1:500) as well as mesenchymal cell marker vimentin (1:10,000; Abcam, Cambridge, MA; #ab11256) and epithelial cell marker E-cadherin (1:1000; Cell Signaling Technology, Danvers, MA; #3195S).

To study the effect and regulation of 15(S)-HETE, amnion fibroblasts were cultured for 3 days before reagent treatment in phenol red- and FBS-free DMEM. To study the effect of 15(S)-HETE on COX-2 expression, time course and concentration-dependent studies were conducted. For time course study, amnion fibroblasts were treated with 15(S)-HETE (10 ng/ml; Cayman) for 1, 3, 6, 12, and 24 h. For concentration-dependent study, amnion fibroblasts were treated with 15(S)-HETE for 24 h at concentrations of 1, 10, and 100 ng/ml. To examine the effect of 15(S)-HETE on the induction of COX-2, microsomal prostaglandin E synthase-1 (mPGES-1), the terminal inducible enzyme responsible for the conversion of PGH2 to PGE2, and PGE2 by proinflammatory mediators in amnion fibroblasts, amnion fibroblasts were treated with lipopolysaccharide (LPS) (10 ng/ml; Sigma), interleukin-1β (IL-1β) (1 ng/ml; Sigma), or acute-phase protein serum amyloid A1 (SAA1) (50 ng/ml; PeproTech Inc, Rocky Hill, NJ) in the presence or absence of 15(S)-HETE (10 ng/ml) for 24 h. To examine the effect of 15(S)-HETE on LPS-, IL-1β-, and SAA1-induced phosphorylation of p65, a subunit of NF-κB, amnion fibroblasts were treated with LPS (10 ng/ml), IL-1β (1 ng/ml), or SAA1 (50 ng/ml) in the presence or absence of 15(S)-HETE (10 ng/ml) for 1, 3, and 6 h. To determine the role of NF-κB in the enhancement of LPS-, IL-1β-, and SAA1-induced COX-2 expression by 15(S)-HETE, the cells were treated with LPS (10 ng/ml), IL-1β (1 ng/ml), or SAA1 (50 ng/ml) for 24 h in the presence or absence of 15(S)-HETE (10 ng/ml) with or without siRNA-mediated knockdown of *RELA* (p65). The method of siRNA transfection is described below. To explore whether PGE2 regulates ALOX15 and ALOX15B expression and 15(S)-HETE production, amnion fibroblasts were treated with PGE2 (0.01–1 μM; Sigma) for 6 h in the presence or absence of EP2 receptor antagonist PF-04418948 (PF; 10 μM; Selleck, Houston, TX) or PKA inhibitor PKI 14–22 amide (PKI; 5 μM; Selleck).

The mRNA abundance of *ALOX15*, *ALOX15B*, *PTGS2* (COX-2), *PTGES* (mPGES-1), *RELA* (p65), and *GAPDH* in amnion fibroblasts was measured with qRT-PCR with primer sequences given in supplemental Table S4. The protein abundance of ALOX15, ALOX15B, COX-2, total p65, phosphorylated p65 at Ser536, and GAPDH in amnion fibroblasts was determined with Western blotting using antibodies against ALOX15 (1:500) and ALOX15B (1:500), COX-2 (1:1000; Cell Signaling; #12282S), total p65 (1:1000; Cell Signaling; #6956S), phosphorylated p65 (Ser536) (1:1000; Cell Signaling; #3033S), and GAPDH (1:10,000). PGE2 and 15(S)-HETE in the culture medium of treated amnion fibroblasts were measured with ELISA kits (Cayman). Detailed methods of qRT-PCR, Western blotting, and ELISA were described above for amnion tissue.

### Transfection of siRNA in human amnion fibroblasts with electroporation

Small interference RNA-mediated knockdown of p65 was used to investigate the role of NF-κB in the enhancement of LPS-, IL-1β-, and SAA1-induced COX-2 expression. Cells were transfected immediately after isolation with 50 nM of siRNA (5′-GCCCUAUCCCUUUACGUCATT-3′) (GenePharma, Shanghai, China) against *RELA* in Opti-MEM (Life Technologies Inc) using an electroporator (Nepa Gene, Chiba, Japan) at 165V for 5 ms. For negative control, randomly scrambled siRNA (5′-UUCUCCGAACGUGUCACGUTT-3′) was used. The cells were then incubated in DMEM containing 10% FBS and antibiotics for three days before treatments with proinflammatory mediators and 15(S)-HETE. The knockdown efficiency of *RELA* was about 90% (supplemental Fig. S1).

### Mouse model to study the role of 15(S)-HETE in parturition

Mouse experimentation was conducted following ARRIVE guidelines, which was approved by the Institutional Review Board of Ren Ji Hospital, Shanghai Jiao Tong University School of Medicine. C57BL/6 mice (Charles River, Beijing, China) aging from 10 to 13 weeks were mated overnight. When a vaginal plug was present, it was counted as 0.5-days post-coitus (dpc). Because *Alox8* is the mouse homolog of human *ALOX15B* gene, which catalyzes the formation of 8(S)-HETE rather than 15(S)-HETE, *Alox15*-encoded ALOX15 is the only enzyme catalyzing the formation of 15(S)-HETE in the mouse [25]. Therefore, only the distribution of ALOX15 was examined in the mouse placenta and fetal membranes (16.5 dpc) with immunohistochemical staining of the paraffin-embedded tissue section with a primary antibody against mouse ALOX15 (1:100; Abcam; #ab244205) following the same protocol as described for human amnion tissue section staining. To study the gestational changes of ALOX15, COX-2, and 15(S)-HETE abundance in the mouse placenta and fetal membranes, the tissues were collected on 14.5, 16.5, and 18.5 dpc. The abundance of ALOX15 and COX-2 in the tissue was determined with Western blotting using antibodies against mouse ALOX15 (1:500; Abcam; #ab244205) and COX-2 (1:1000; Cell Signaling; #12282S) after protein extraction with the RIPA lysis buffer. The abundance of 15(S)-HETE in the tissue was measured with an ELISA kit (Cayman) after extraction with ethyl acetate.

To observe whether 15(S)-HETE could induce preterm birth, 15(S)-HETE (50 μg) or an equal volume of solvent was injected subcutaneously every 12 h on 16.5, 17, and 17.5 dpc. Some of the mice (n = 18) were allowed to deliver spontaneously for observation of delivery time, and some (n = 14) were sacrificed on 18 dpc for collection of placentae and fetal membranes to examine the effect of 15(S)-HETE administration on the abundance of COX-2 and PGE2 in the tissue. The abundance of COX-2 was measured with Western blotting with the antibody described above after protein extraction with the RIPA lysis buffer, and PGE2 concentration in the tissue was determined with an ELISA kit (Cayman) after extraction with ethyl acetate. Detailed methods of Western blotting and ELISA were described for human amnion tissue.

### Statistical analysis

All data are presented as means ±SEM. The number of repeated experiments in each study was separate experiments reflecting independent patients or animals. After normality testing with the Shapiro-Wilk test, paired or unpaired Student’s *t*-test or Mann-Whitney U test was used to compare two groups. One-way ANOVA followed by Newman-Keuls multiple-comparisons test was performed when assessing the differences among multiple groups. Pearson correlation analysis was performed to test the correlation between PGE2 and 15(S)-HETE levels. Fisher exact tests were applied to compare the preterm birth rates in the mice study. Statistical significance was defined as *P* < 0.05.

## Results

### Identification of eicosanoids pertinent to parturition in human amnion with AA-targeted metabolomics

To screen AA-derived eicosanoids which were altered in the amnion at parturition, LC-MS-based metabolomics targeted for AA-derived eicosanoids was performed on human amnion tissue obtained from TL (n = 10) and TNL (n = 8). The demographic and clinical characteristics of recruited women for this study are given in supplemental Table S1. After quality control (supplemental Fig. S2), ten major AA-derived eicosanoids were detected in the amnion tissue (supplemental Table S5). Principal component analysis revealed that the abundance of AA-derived eicosanoids in TL and TNL groups was clustered distinctively (Fig. 1B), suggesting that labor was associated with a distinct profile of alteration in AA-derived eicosanoids in human amnion. Specifically, three eicosanoids (PGE2, PGF2α, and 15-HETE) were significantly increased and two eicosanoids (AA and thromboxane B2) were significantly decreased in TL group when compared to those in TNL group (Fig. 1C, D). Our findings confirmed the depletion of AA, increased PGE2 and PGF2α production, and decreased thromboxane B2 abundance in the amnion in labor at term (22, 30, 31, 32). We also confirmed that the amnion produced much more PGE2 than PGF2α in both labor and nonlabor status (Fig. 1D) (10, 24, 33). Although several previous studies have demonstrated that 15-HETE levels are increased in amniotic fluid and maternal blood in both term and preterm birth (34, 35, 36, 37), we revealed for the first time that 15-HETE abundance was increased in the amnion at parturition. Because the exact role of 15-HETE in parturition has never been specified, we subsequently focused on 15-HETE to further define its role and synthetic regulation in parturition.

### Increased 15(S)-HETE abundance in human amnion in TL and PL

**Fig. 2:**
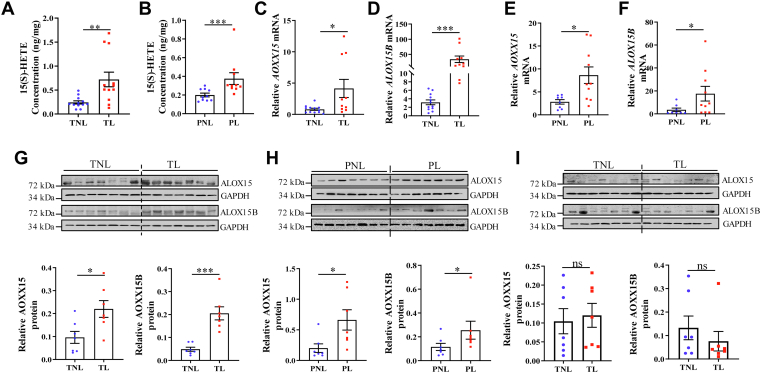
Increased abundance of 15(S)-HETE, ALOX15, and ALOX15B in human amnion in term and preterm birth. A: 15(S)-HETE abundance in the amnion obtained from term delivery with labor (TL, n = 14) and without labor (TNL, n = 14) as measured with ELISA. B: 15(S)-HETE abundance in the amnion obtained from preterm delivery with labor (PL, n = 10) and without labor (PNL, n = 10) as measured with ELISA. C and D: Abundance of *ALOX15* and *ALOX15B* mRNA in the amnion collected from TL (n = 10) and TNL (n = 12) as measured with qRT-PCR. E and F: Abundance of *ALOX15* and *ALOX15B* mRNA in the amnion collected from PL (n = 10) and PNL (n = 8) as measured with qRT-PCR. G: Abundance of ALOX15 and ALOX15B protein in the amnion collected from TL (n = 7) and TNL (n = 7) as measured with Western blotting. H: Abundance of ALOX15 and ALOX15B protein in the amnion collected from PL (n = 7) and PNL (n = 7) as measured with Western blotting. I: Abundance of ALOX15 and ALOX15B protein in the chorion/decidua layer collected from TL (n = 7) and TNL (n = 7) as measured with Western blotting. Statistical analysis was performed with unpaired Student’s *t*-test (E and G) or Mann-Whitney U test (A–D, F, H–I). ns, no significant. ∗*P*<0.05, ∗∗*P*<0.01, ∗∗∗*P*<0.001 versus TNL or PNL. ALOX15, lipoxygenase 15; ALOX15B, lipoxygenase 15B; PNL, preterm nonlabor; qRT-PCR, quantitative real-time polymerase chain reaction; TNL, term nonlabor; TL, term labor.

Two isomers of 15-HETE, 15(S)-HETE, and 15(R)-HETE exist in the body, but the ALOX pathway produces only 15(S)-HETE but not 15(R)-HETE (25, 26, 27, 28, 38). Next, we collected additional human amnion tissue from both term and preterm deliveries to measure the changes of 15(S)-HETE with labor at term and preterm with ELISA. The demographic and clinical characteristics of recruited women for this study are given in supplemental Tables S2 and S3. Results showed that 15(S)-HETE abundance was significantly increased not only in TL group (n = 14) but also in PL group (n = 10) when compared to those in TNL group (n = 14) and PNL group (n = 10), respectively (Fig. 2A, B), suggesting that increased 15(S)-HETE synthesis may be associated with both TL and PL.

### Increased ALOX15 and 15B abundance in human amnion in TL and PL

It is known that 15(S)-HETE is formed from AA through the ALOX pathway under the catalysis of ALOX15 and 15B in humans (Fig. 1A) (27, 28). We next examined whether the abundance of ALOX15 and 15B mRNA and protein was consistently altered in human amnion in TL and PL. Analyses with qRT-PCR and Western blotting showed that the abundance of ALOX15 and 15B mRNA and protein was significantly increased in the amnion in both TL and PL groups when compared to those in TNL and PNL groups, respectively (Fig. 2C–H). Interestingly, ALOX15 and 15B protein were hardly detectable in human placenta. Although ALOX15 and 15B were detectable in the chorion/decidua layer of the fetal membranes, but they displayed no significant changes in TL group (Fig. 2I). These data suggest that increased 15(S)-HETE synthesis occurs mainly in the amnion in human parturition.

### Distribution of ALOX15 and 15B in human amnion

**Fig. 3:**
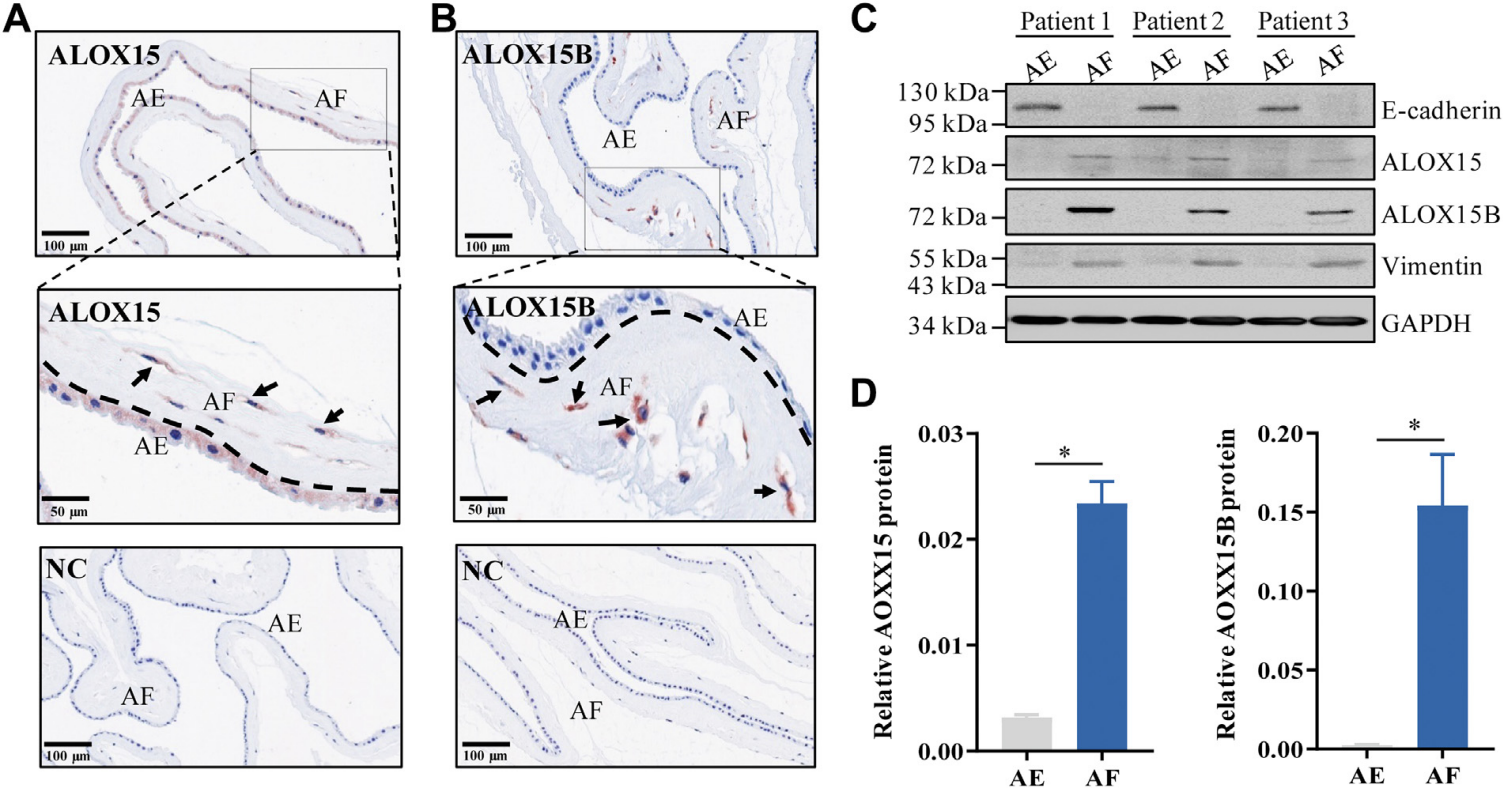
Distribution of ALOX15 and ALOX15B in human amnion. A and B: Representative immunohistochemical images showing the distribution of ALOX15 and ALOX15B in human amnion obtained from term deliveries without labor (TNL). n = 3. C and D: Western blotting analysis showing ALOX15 and ALOX15B abundance in cultured amnion epithelial cells and fibroblasts prepared from three individual subjects of the TNL group. E-cadherin and vimentin served as markers for epithelial cells and fibroblasts, respectively. Statistical analysis was performed with paired Student’s *t* test. ∗*P*<0.05 versus AE. AE, amnion epithelial cells; AF, amnion fibroblasts; ALOX15, lipoxygenase 15; ALOX15B, lipoxygenase 15B; NC, negative control; TNL, term nonlabor.

Immunohistochemical staining showed that ALOX15B distributed almost exclusively in the mesenchymal fibroblasts of human amnion, while ALOX15 appeared to distribute in both mesenchymal fibroblasts and epithelial cells (Fig. 3A, B). Western blotting showed that both ALOX15 and 15B were much more abundant in amnion fibroblasts than in epithelial cells (Fig. 3C, D). In epithelial cells, ALOX15B protein was hardly detectable, while low level of ALOX15 protein was detected (Fig. 3C). Since mesenchymal fibroblasts have also been shown to be the primary site of PGE2 synthesis in human amnion (39), we next used primary human amnion fibroblasts to examine the effect of 15(S)-HETE on PGE2 synthesis.

### Effect of 15(S)-HETE on COX-2 expression and PGE2 production in human amnion fibroblasts

**Fig. 4:**
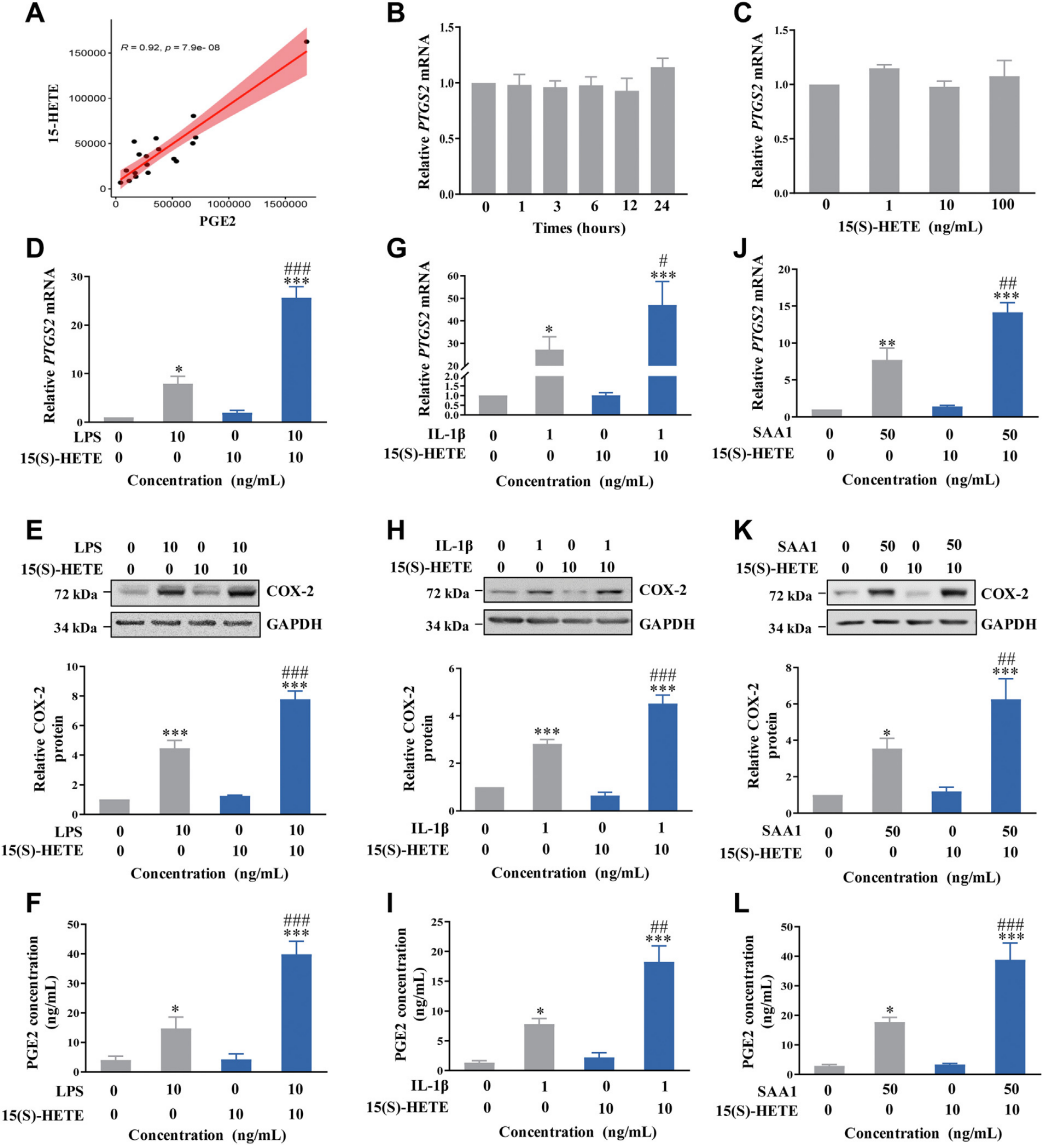
Amplification of proinflammatory mediators-induced COX-2 expression and PGE2 production by 15(S)-HETE in human amnion fibroblasts. A: Pearson analysis showing the correlation between 15-HETE and PGE2 levels in the amnion as measured with AA-targeted metabolomics (n = 18). B: Time course study of the effect of 15(S)-HETE (10 ng/ml; 1, 3, 6, 12, and 24 h) on *PTGS2* mRNA expression. n = 3. C: Concentration-dependent study of the effect of 15(S)-HETE (1, 10, and 100 ng/ml; 24 h) on *PTGS2* mRNA expression. n = 3. D–F: Effect of 15(S)-HETE (10 ng/ml) on LPS (10 ng/ml; 24 h)-induced *PTGS2* mRNA (D, n = 3) and COX-2 protein (E, n = 4) expression and PGE2 production (F, n = 4). G–I: Effect of 15(S)-HETE (10 ng/ml) on IL-1β (1 ng/ml; 24 h)-induced *PTGS2* mRNA (G, n = 4) and COX-2 protein (H, n = 4) expression and PGE2 production (I, n = 5). J–L: Effect of 15(S)-HETE (10 ng/ml) on SAA1 (50 ng/ml; 24 h)-induced *PTGS2* mRNA (J, n = 3) and COX-2 protein (K, n = 4) expression and PGE2 production (L, n = 4). Statistical analysis was performed with one-way ANOVA test followed by Newman-Keuls multiple-comparisons test. Top panels of E, H, and K are the representative immunoblots. ∗*P*<0.05, ∗∗*P*<0.01, ∗∗∗*P*<0.001 versus control (without treatment). #*P*<0.05, ##*P*<0.01, ###*P*<0.001 versus LPS, IL-1β or SAA1 group. AA, arachidonic acid; COX-2, cyclooxygenase-2; IL-1β, interleukin-1β; LPS, lipopolysaccharide; PGE2, prostaglandin E2; SAA1, serum amyloid A1.

Pearson analysis showed a significantly positive correlation between 15(S)-HETE and PGE2 levels in the amnion in AA-targeted metabolomics study (R = 0.92, *P* = 7.9e-8) (Fig. 4A), indicating that synthesis of 15(S)-HETE and PGE2 may be associated in the amnion. Unexpectedly, we failed to observe any effect of 15(S)-HETE per se on COX-2 expression in amnion fibroblasts in either time course (10 ng/ml; 1, 3, 6, 12, and 24 h) or concentration-dependent (1, 10, and 100 ng/ml; 24 h) studies (Fig. 4B, C). Surprisingly, we found that 15(S)-HETE (10 ng/ml; 24 h) potently bolstered the induction of *PTGS2* mRNA and COX-2 protein expression and PGE2 production by proinflammatory mediators including LPS (10 ng/ml; 24 h) (Fig. 4D–F), IL-1β (1 ng/ml; 24 h) (Fig. 4G–I), and SAA1 (50 ng/ml; 24 h) (Fig. 4J–L) despite the ineffectiveness of 15(S)-HETE on its own (Fig. 4D–L). In addition, we found that 15(S)-HETE (10 ng/ml; 24 h) also enhanced LPS (10 ng/ml; 24 h)-, IL-1β (1 ng/ml; 24 h)-, and SAA1 (50 ng/ml; 24 h)-induced mPGES-1 expression in human amnion fibroblasts (supplemental Fig. S3), suggesting that 15(S)-HETE might potentiate PGE2 production by enhancing the induction of both COX-2 and mPGES-1 expression by proinflammatory mediators in human amnion fibroblasts.

### Involvement of NF-κB in the enhancement of inflammatory mediators-induced COX-2 expression by 15(S)-HETE in human amnion fibroblasts

**Fig. 5:**
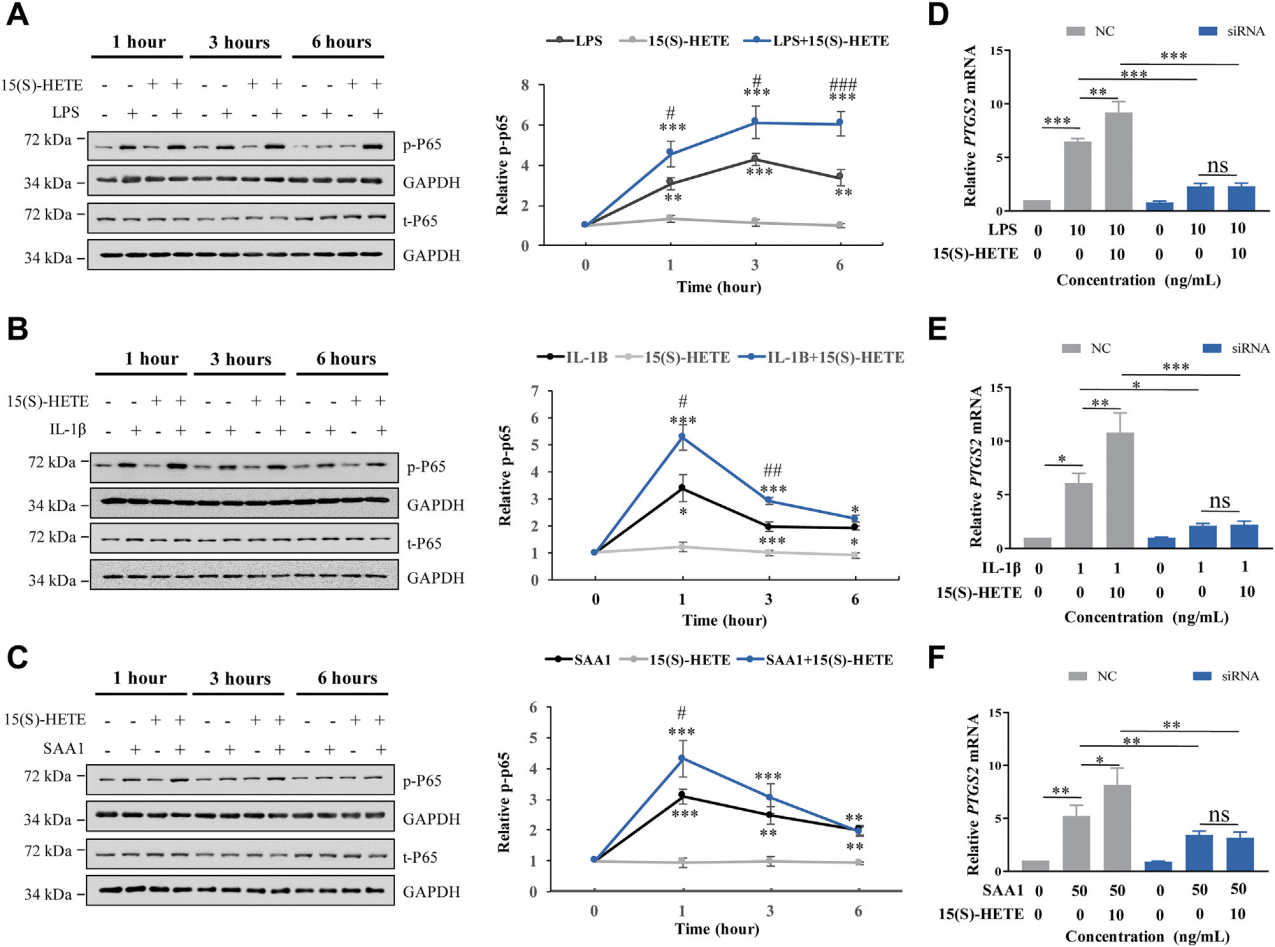
Amplification of proinflammatory mediators-induced phosphorylation of p65 by 15(S)-HETE in human amnion fibroblasts. A–C: Effect of 15(S)-HETE (10 ng/ml; 1, 3, 6 h) on LPS (10 ng/ml; A)-, IL-1β (1 ng/ml; B)-, and SAA1 (50 ng/ml; C)-induced p65 phosphorylation. n = 4. ∗*P*<0.05, ∗∗*P*<0.01, ∗∗∗*P*<0.001 versus control (0 h). #*P*<0.05, ##*P*<0.01, ###*P*<0.001 versus IL-1β, LPS, or SAA1 group. D–F: Effect of siRNA-mediated knockdown of p65 on the induction of *PTGS2* mRNA expression by LPS (10 ng/ml; 24 h; D), IL-1β (1 ng/ml; 24 h; E), or SAA1 (50 ng/ml 24 h; F) in the presence or absence of 15(S)-HETE (10 ng/ml). n = 3. ∗*P*<0.05, ∗∗*P*<0.01, ∗∗∗*P*<0.001. ns, no significant. Statistical analysis was performed with one-way ANOVA test followed by Newman-Keuls multiple-comparisons test. Left panels of A–C are the representative immunoblots. SAA1, serum amyloid A1; LPS, lipopolysaccharide; IL-1β, interleukin-1β.

NF-κB is a classical proinflammatory transcription factor mediating the expression of a wide array of proinflammatory mediators including COX-2 (19, 20). Thus, we explored the role of NF-κB in the enhancement of proinflammatory mediators-induced COX-2 expression by 15(S)-HETE in human amnion fibroblasts. Activation of NF-κB was determined by examining the phosphorylation of the p65 subunit of NF-κB. Consistently, 15(S)-HETE (10 ng/ml) failed to affect p65 phosphorylation on its own, but it significantly enhanced the phosphorylation of p65 by LPS (10 ng/ml) at 1, 3, and 6 h, IL-1β (1 ng/ml) at 1 and 3 h, and SAA1 (50 ng/ml) at 1 h in human amnion fibroblasts (Fig. 5A–C). Correspondingly, siRNA-mediated knockdown of *RELA* (P65) significantly attenuated the induction of COX-2 expression not only by LPS (10 ng/ml), IL-1β (1 ng/ml), and SAA1 (50 ng/ml) themselves but also the potentiated induction by 15(S)-HETE (10 ng/ml) (Fig. 5D–F).

### PGE2 is an upstream stimulator of ALOX15 and 15B expression and 15(S)-HETE production in human amnion fibroblasts

**Fig. 6:**
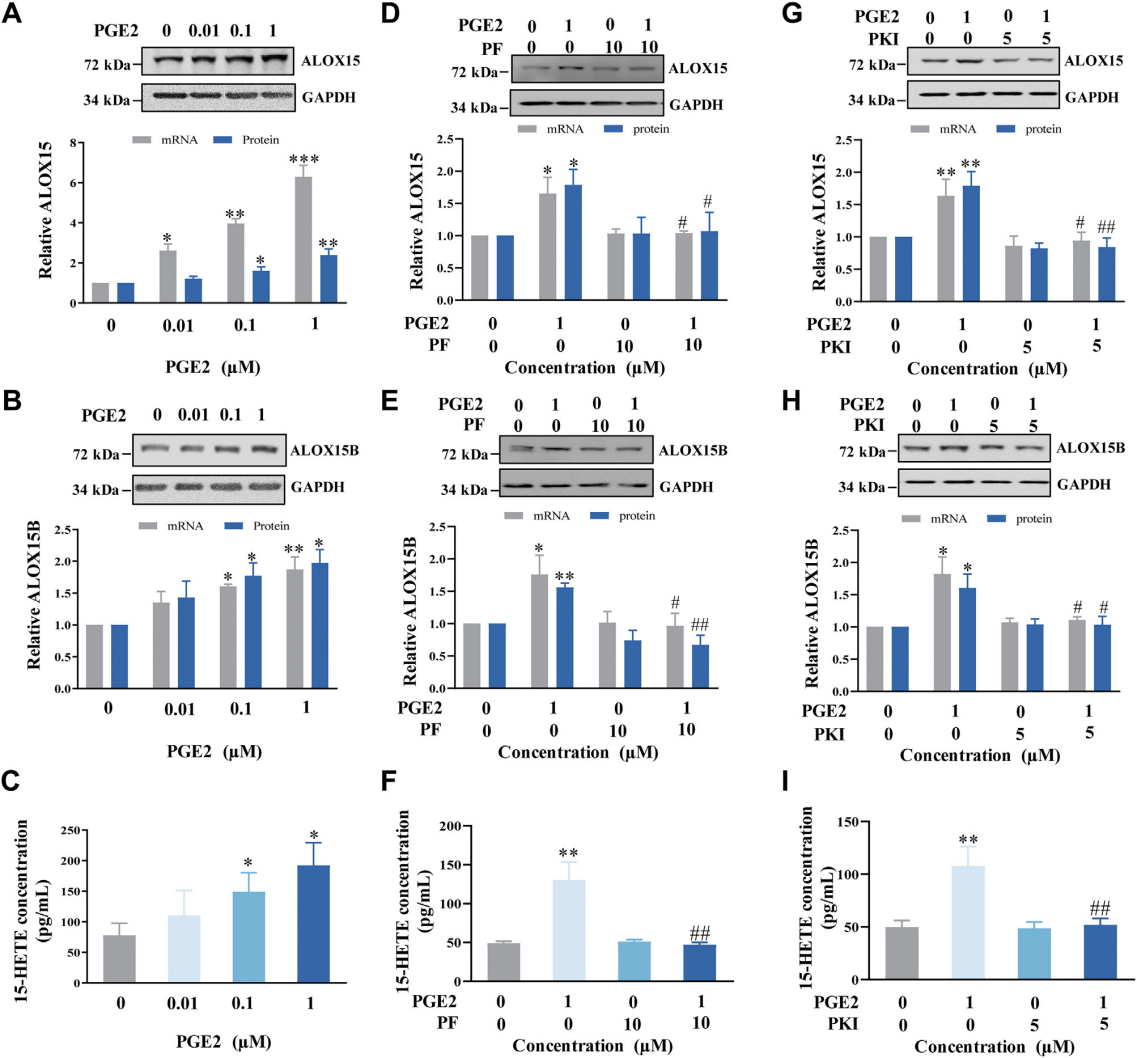
Effect of PGE2 on ALOX15 and ALOX15B expression in human amnion fibroblasts. A: Concentration-dependent induction of *ALOX15* mRNA (n = 3) and protein (n = 5) expression by PGE2 (0.01, 0.1, and 1 μM; 6 h). B: Concentration-dependent induction of *ALOX15B* mRNA (n = 3) and protein (n = 4) expression by PGE2 (0.01, 0.1, and 1 μM; 6 h) in amnion fibroblasts. C: Concentration-dependent induction of 15(S)-HETE production by PGE2 (0.01, 0.1, and 1 μM; 6 h) in amnion fibroblasts. n = 3. D–F: EP2 receptor antagonist PF-04418948 (PF; 10 μM) blocked PGE2 (1 μM; 6 h)-induced ALOX15 (D) and ALOX15B (E) mRNA and protein expression and 15(S)-HETE production (F). n = 3. G–I: PKA inhibitor PKI 14–22 amide (PKI; 5 μM) blocked PGE2 (1 μM; 6 h)-induced ALOX15 (G) and ALOX15B (H) mRNA and protein expression and 15(S)-HETE production (I) in amnion fibroblasts. n = 3. Statistical analysis was performed with one-way ANOVA test followed by Newman-Keuls multiple-comparisons test. Top panels of A, B, D, E, G, and H are the representative immunoblots. ∗*P*<0.05, ∗∗*P*<0.01, ∗∗∗*P*<0.001 versus control (without treatment). #*P*<0.05, ##*P*<0.01 versus PGE2 group. ALOX15, lipoxygenase 15; ALOX15B, lipoxygenase 15B; PGE2, prostaglandin E2.

Our data presented above indicate that 15(S)-HETE derived from the ALOX15/15B pathway may be an amplifier of COX-2 expression and PGE2 production in human amnion fibroblasts in the inflammatory process of parturition. Given the self-reinforcing role of PGE2 in membrane activation through induction of its own synthetic enzyme COX-2 expression in human amnion fibroblasts (40), we postulated that PGE2 may also be a stimulator of ALOX15 and 15B expression. We found that PGE2 (0.01–1 μM; 6 h) increased ALOX15 and 15B mRNA and protein abundance in amnion fibroblasts in a concentration-dependent manner with concomitant increased 15(S)-HETE production (Fig. 6A–C), which was blocked by either a PGE2 EP2 receptor antagonist PF-04418948 (10 μM) or a PKA inhibitor PKI 14–22 amide (5 μM) (Fig. 6 D–I). These data suggest that PGE2 and 15(S)-HETE may form a feed-forward loop in amnion fibroblasts in the inflammatory process of parturition.

### Induction of preterm birth by 15(S)-HETE in the mouse

**Fig. 7:**
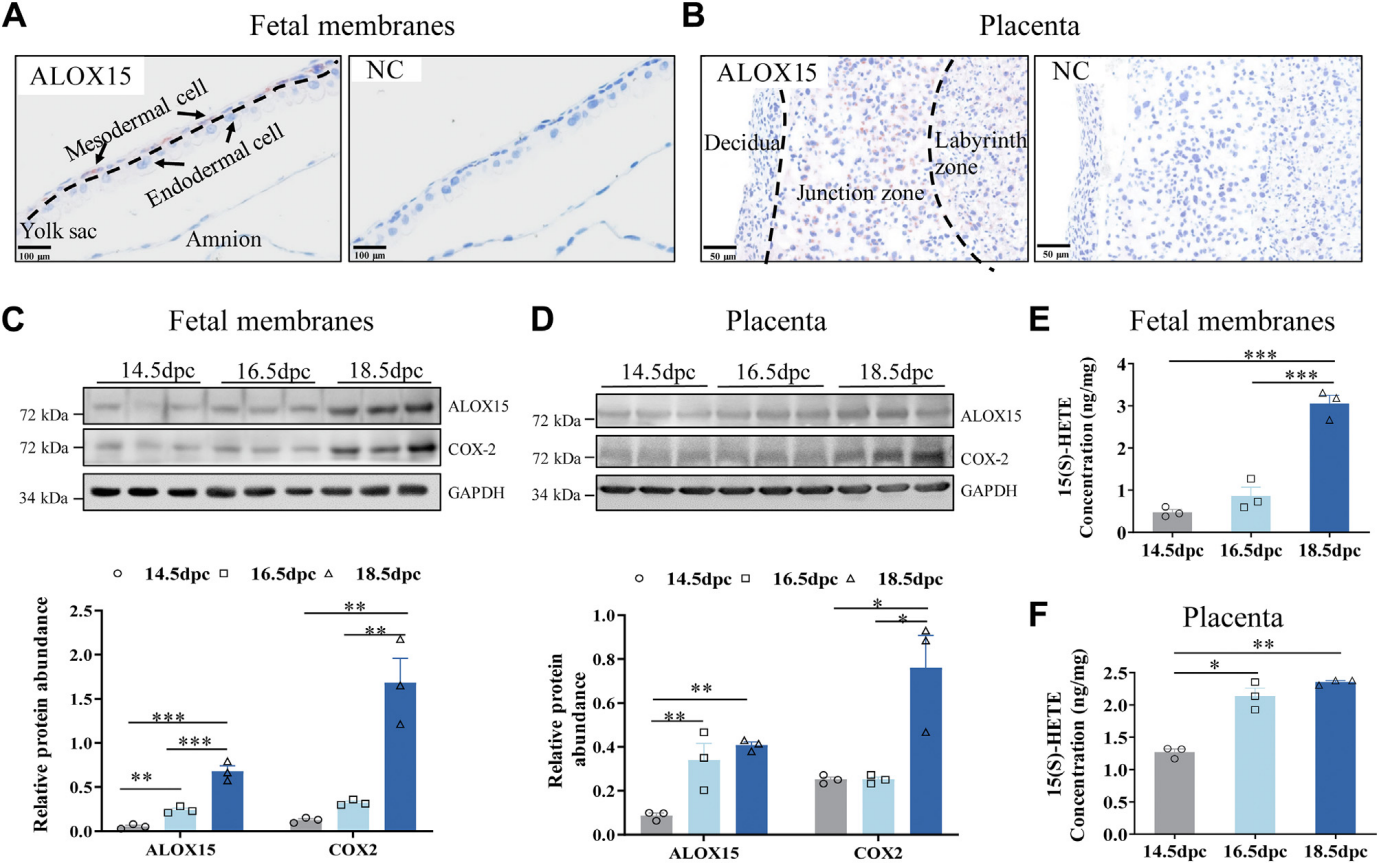
Gestational age-dependent changes of ALOX15 and 15(S)-HETE abundance in mouse fetal membranes and placenta. A and B: Representative immunohistochemical images showing the distribution of ALOX15 in mouse fetal membranes (A) and placenta (B) on dpc 16.5. n = 3. C and D: Western blotting analysis showing increased ALOX15 and COX-2 protein abundance with gestational age from 14.5 to 18.5 dpc in mouse fetal membranes (C) and placenta (D). E and F: ELISA analysis showing increased 15(S)-HETE abundance with gestational age from dpc 14.5 to dpc 18.5 in mouse fetal membranes (E) and placenta (F). Statistical analysis was performed with one-way ANOVA test followed by Newman-Keuls multiple-comparisons test (C–F). ∗*P*<0.05, ∗∗*P*<0.01, ∗∗∗*P*<0.001. ALOX15, lipoxygenase 15; COX-2, cyclooxygenase-2; dpc, days post-coitus; NC, negative control.

**Fig. 8:**
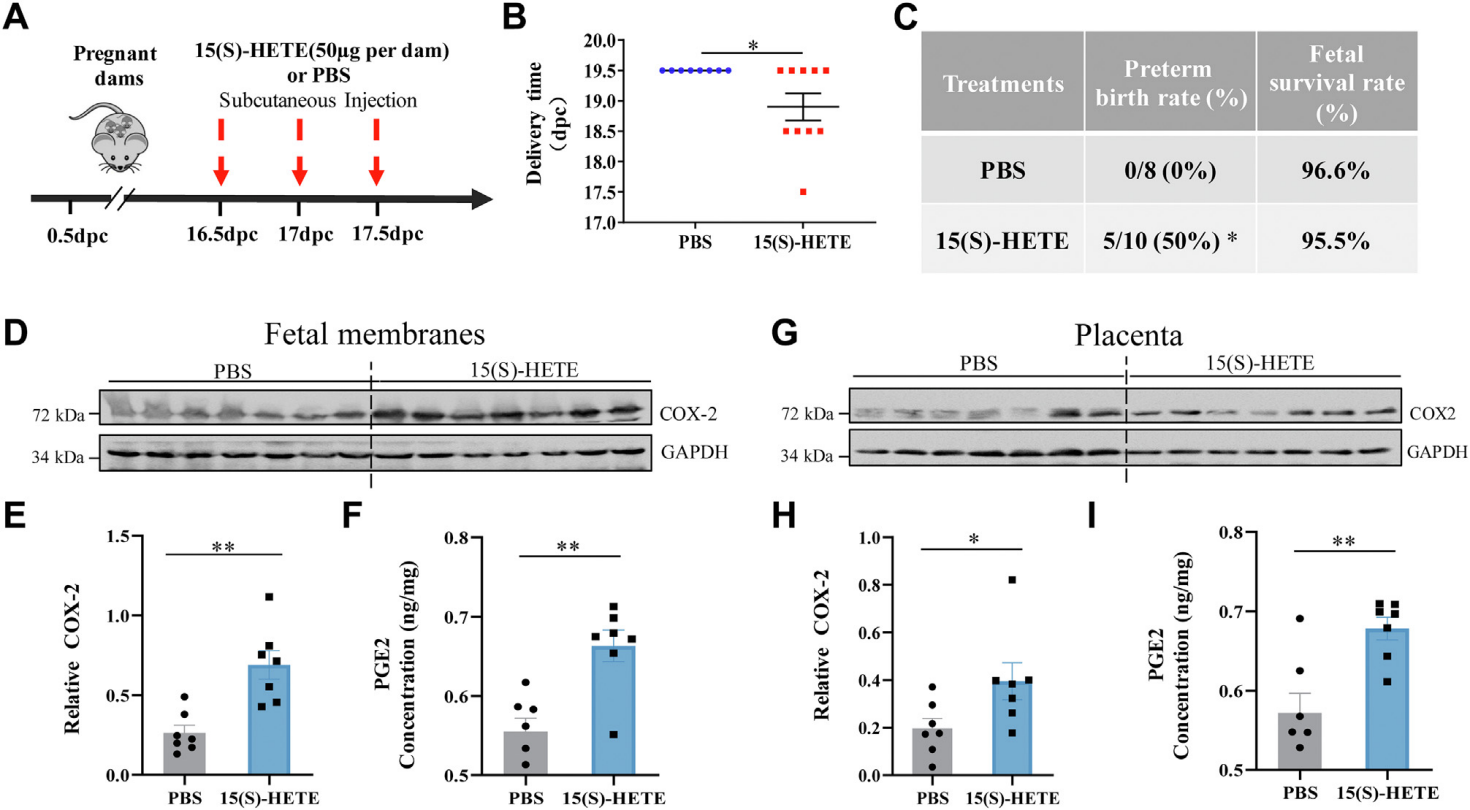
15(S)-HETE-induced preterm birth in mice. A: Time-line illustrating the procedure of 15(S)-HETE administration in pregnant mice. B and C: Subcutaneous injection of 15(S)-HETE (50 μg per dam; every 12 h on 16.5, 17, and 17.5 dpc) induced preterm birth (n = 10). Control mice was given an equal volume of PBS (n = 8). D–L: 15(S)-HETE administration (50 μg per dam; every 12 h on 16.5, 17, and 17.5 dpc) increased COX-2 expression and PGE2 concentration in mouse fetal membranes (D–F) and placenta (G–L) (n = 7). Control mice received an equal volume of PBS (n = 7). Statistical analysis was performed with unpaired Student’s *t* test (B, D, E, G, and H) or Fisher exact test (C). ∗*P*<0.05, ∗∗*P*<0.01 versus PBS group. COX-2, cyclooxygenase-2; dpc, days post-coitus; PGE2, prostaglandin-2.

Evidence gathered in human studies indicates a possible pivotal role of 15(S)-HETE in the initiation of parturition, which prompted us to investigate whether administration of 15(S)-HETE could indeed induce preterm birth in mice. Given that ALOX15 is the only enzyme catalyzing the formation of 15(S)-HETE in mice, we first examined the expressional profile of ALOX15 in the mouse placenta and fetal membranes, both of which are important contributors of PGs in parturition in the mouse (41, 42). Immunohistochemical staining showed that ALOX15 was present both in the yolk sac mesodermal cells of the fetal membranes and in the junctional zone of the placenta (Fig. 7A, B), which contrasted to the hardly detectable levels of ALOX15 and 15B in human placenta. Moreover, the abundance of 15(S)-HETE, ALOX15, and COX-2 in the mouse placenta and fetal membranes increased in a gestational age-dependent manner from 14.5 to 18.5 dpc (Fig. 7C–F). Notably, subcutaneous injection of 15(S)-HETE (50 μg; once half day) on 16.5, 17, and 17.5 dpc induced preterm birth by 0.5–2 days (Fig. 8A–C), along with increased COX-2 and PGE2 abundance in the placenta and fetal membranes (Fig. 8D–H).

## Discussion

In this study, we have provided evidence for the first time that 15(S)-HETE synthesized in human amnion plays a pivotal role in the initiation of parturition by bolstering the activation of NF-κB by proinflammatory mediators including LPS, IL-1β, and SAA1 with consequently amplified COX-2 expression and PGE2 production in amnion fibroblasts. Given parturition being a process of inflammation of gestational tissues (17, 18, 43) and the important role of LPS, IL-1β, and SAA1 in infection and noninfection-induced inflammatory reaction (14, 44), our findings may be fitted into both normal and infection-induced parturition. The important role of 15(S)-HETE in parturition was endorsed by increased ALOX15/15B and 15(S)-HETE abundance in human amnion in spontaneous TL and PL as well as by the finding that 15(S)-HETE administration induced preterm birth in the mouse.

Previous studies have demonstrated that 15(S)-HETE might be associated with several events in reproduction, including ovulation, embryo implantation, pre-eclampsia, etc. (45, 46, 47, 48). Increased 15(S)-HETE levels have been found in amniotic fluid and maternal blood in both term and preterm birth (34, 35, 36, 37). However, neither the source nor the role of 15(S)-HETE in parturition has ever been specified. An early study investigated AA metabolism by ALOX pathways in human intrauterine tissues and found that none of AA metabolites showed significant changes with labor in rates of formation, including 15-HETE in the amnion (49). However, in this study, we found that the amnion expressed much more ALOX15/15B than the chorion/decidua and placenta, and, moreover, only the amnion but not the chorion/decidua and placenta exhibited significant increases in ALOX15/15B and 15(S)-HETE abundance at parturition, suggesting that increased 15(S)-HETE levels in amniotic fluid in parturition may reflect the increased synthesis of 15(S)-HETE in the amnion. The human amnion is known to be particularly rich in AA content, which is greatly depleted during parturition (22). Considering that PGE2, PGF2α, and 15(S)-HETE are the only three eicosanoids increased in the amnion in parturition as revealed by AA-targeted metabolomics in this study, we believe that AA is mobilized in the amnion mainly for the synthesis of PGE2, PGF2α, and 15(S)-HETE in parturition.

In this study, we demonstrated that 15(S)-HETE promoted parturition through potentiation of the induction of COX-2 expression and PGE2 production by proinflammatory mediators as illustrated in both human amnion fibroblasts and mice studies. Our findings coincidently reconciled the findings in colonic myofibroblasts and follicular dendritic cell-like cells that 15(S)-HETE also enhances COX-2 expression by IL-1β (50, 51). COX-2 is highly inducible by proinflammatory mediators at sites of inflammation, which is responsible for the production of abundant PGs in inflammation. PGs produced in gestational tissues, specifically PGE2 and PGF2α, are conferred with specific parturition-pertinent effects, that is, stimulation of myometrial contraction and cervical ripening as well as membrane activation (11, 12, 13). As such, the regulatory mechanism underlying COX-2 expression in gestational tissues has been intensively investigated. It is known that the expression of COX-2 is under the tight control of the classical proinflammatory transcription factor NF-κB (52, 53). Here in this study, we also showed that 15(S)-HETE bolstered the induction of COX-2 expression by proinflammatory mediators though potentiation of NF-κB activation in human amnion fibroblasts. Considering that the classical role NF-κB in mediation of the expression of a wide array of proinflammatory mediators in inflammation, we speculate that there may be other inflammatory mediators whose expression can be amplified by 15(S)-HETE in the amnion fibroblasts during the inflammatory process of parturition. Undoubtedly, it should be an interesting issue to explore with in the future.

A number of potential receptors have been suggested for 15(S)-HETE including peroxisome proliferator-activated receptor γ (PPARγ), leukotriene B4 type-2 receptor, and transient receptor potential vanilloid subfamily member 1 (54, 55, 56). PPARγ has been reported to be a receptor for 15(S)-HETE in macrophages (57). However, concomitantly, opposite changes in PPARγ and COX-2 have been reported in the fetal membranes at the onset of labor (58), suggesting that PPARγ is unlikely a receptor mediating the effect of 15(S)-HETE in the amnion. We are unclear at the current stage which putative receptor mediates the amplification of proinflammatory mediators-induced NF-κB activation by 15(S)-HETE in human amnion fibroblasts.

Notably, we discovered in this study that PGE2 was a stimulator of ALOX15 and 15B expression in human amnion fibroblasts. This finding is worth particularly emphasizing because the induction of ALOX15 and 15B by PGE2 filled a gap in the feed-forward loop between 15(S)-HETE and PGE2 production in amnion fibroblasts. We believe that this feed-forward loop is an important strategy adopted in pregnancy to ensure the adequate production of 15(S)-HETE and PGE2 for parturition, which appears to fit well into the feed-forward mechanism of parturition (59). There are four PGE2 receptors, namely, EP1, 2, 3, and 4 (60). Among them, EP2 and EP4 are more abundantly expressed in human amnion fibroblasts (40). Our recent study has shown that the expression of EP2 increases, whereas the expression of EP4 decreases in the amnion tissue in parturition despite that both receptors are reportedly coupled with the cAMP/PKA pathway (40). Moreover, we have demonstrated that EP2 receptor mediates the induction of COX-2 by PGE2 in amnion fibroblasts, an effect that can be attenuated by the PI3K pathway coupled with the more complicated EP4 receptor (40), which may explain why these two PGE2 receptors manifested opposite changes in the amnion in parturition. Here in this study, we demonstrated that EP2 receptor also mediated the induction of ALOX15 and 15B by PGE2 in amnion fibroblasts, suggesting again that the EP2 receptor plays a crucial role in parturition by mediating the induction of both COX-2 and ALOX15/15B expression in human amnion fibroblasts. Our findings provide further evidence that PGE2 is an important activator of membrane activation resulting in the surges of both PGE2 and 15(S)-HETE syntheses.

In conclusion, 15(S)-HETE synthetized by ALOX15 and ALOX15B plays an important role in parturition by forming a feed-forward loop with the COX-2/PGE2 pathway through potentiation of inflammation-induced NF-κB activation. Interruption of this feed-forward loop may be of therapeutic value for the treatment of preterm birth.

## Data Availability

The original data and materials presented in the study are available from the corresponding authors upon reasonable request.

## Supplemental Data

This article contains supplemental data.

## Conflict of Interest

The authors declare that they have no competing interests.
