# Supplementary material for: Identification of a Feed-Forward Loop Between 15(S)-HETE and PGE2 in Human Amnion at Parturition
Source: J Lipid Res. 2022 Oct 4;63(11):100294. doi: 10.1016/j.jlr.2022.100294 (PMC9646666; doi:10.1016/j.jlr.2022.100294)
Supplement: Supplementary figures and tables [file mmc1.pdf]

Supplementary Materials for

Identification of a feed-forward loop between 15(S)-HETE and PGE2 in human amnion at parturition

Fan Zhang[1,2], Kang Sun[1,2*], Wang-Sheng Wang[1,2*]

Correspondence authors: Dr. Wang-sheng Wang, wangsheng_wang@hotmail.com or Dr. Kang Sun, sungangrenji@hotmail.com

Contents:

Figure S1 The efficiency of siRNA-mediated knockdown of *RELA* in human amnion fibroblasts.

Figure S2 Relative standard deviation percentage of quality control samples for AA-target metabolomics.

Figure S3 Amplification of proinflammatory mediators-induced *PTGES* mRNA expression by 15(S)-HETE in human amnion fibroblasts.

Table S1 Demographic and clinical characteristics of recruited pregnant women at term for AA-targeted metabolomics study.

Table S2 Demographic and clinical characteristics of recruited pregnant women at term for the study with ELISA assay, qRT-PCR and Western blotting.

Table S3 Demographic and clinical characteristics of recruited pregnant women at preterm for the study with ELISA assay, qRT-PCR and Western blotting.

Table S4 Primer sequences used in qRT-PCR.

Table S5 Abundance of AA metabolites in human amnion in TL and TNL.

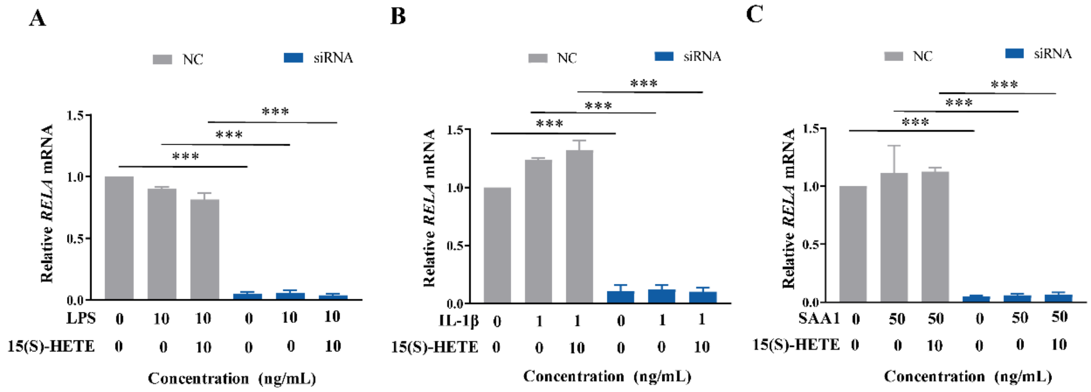

Figure S1 The efficiency of siRNA-mediated knockdown of *RELA* in human amnion fibroblasts. n=3. Data are shown as mean ± SEM. *p<0.05, **p<0.01, ***p<0.001. ns, no significant. Statistical analysis was performed with one-way ANOVA test followed by Newman-Keuls multiple-comparisons test.

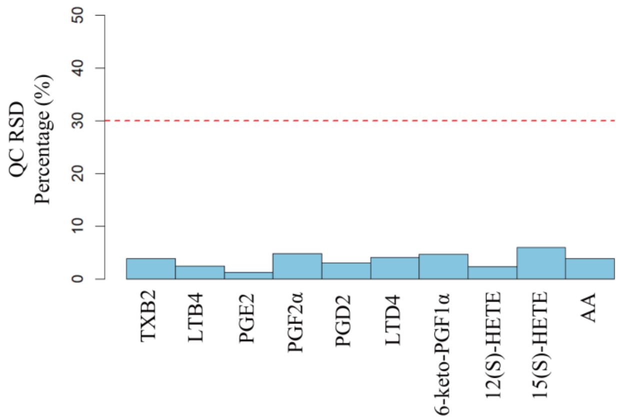

Figure S2 Relative standard deviation (RSD) percentage of quality control (QC) samples for AA-target metabolomics. RSD percentage of QC samples is used for evaluating method repeatability. The cutoff sets as 30%.

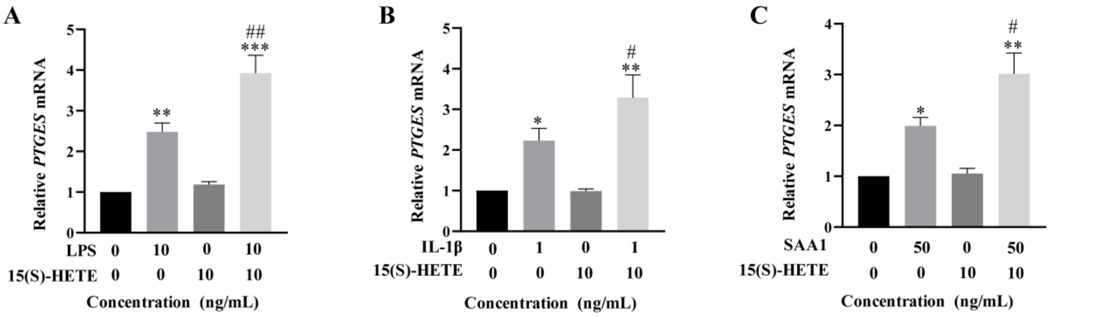

Figure S3 Amplification of proinflammatory mediators-induced *PTGES* mRNA expression by 15(S)-HETE in human amnion fibroblasts. (A) Effect of 15(S)-HETE (10 ng/mL) on LPS (10 ng/mL; 24 hours)-induced *PTGES* mRNA expression. n=3. (B) Effect of 15(S)-HETE (10 ng/mL) on IL-1β (1 ng/mL; 24 hours)-induced *PTGES* mRNA expression. n=3. (C) Effect of 15(S)-HETE (10 ng/mL) on SAA1 (50 ng/mL; 24 hours)-induced *PTGES* mRNA expression. n=3. Data are shown as mean ± SEM. Statistical analysis was performed with one-way ANOVA test followed by Newman-Keuls multiple-comparisons test. *$p<0.05$, **$p<0.01$ vs control (without LPS and 15(S)-HETE treatment). #$p<0.05$, ##$P<0.01$ *vs* LPS, IL-1β or SAA1 treatment.

Table S1. Demographic and clinical characteristics of recruited pregnant women at term for AA-targeted metabolomics study

	**TNL (n=8)**	**TL (n=10)**	***P* value**
Maternal age (year)	30.63±1.21	30.20±0.70	0.76
Gestational age at delivery (week)	38.80±0.18	39.17±0.20	0.19
Delivery mode			
Vaginal (%)	0	100%	N/A
C-section (%)	100%	0	N/A
Membrane rupture			
SPOM (%)	0	100%	N/A
ARM (%)	100%	0	N/A
Gravidity median (range)	2 (1-3)	2 (1-3)	0.30
Parity median (range)	2 (1-2)	1 (1-2)	0.06
Fetal gender (male/female)	4/4	4/6	0.67
Birth weight (g)	3545.63±108.80	3372.5±102.25	0.26

TNL, term not in labor; TL, term in labor; ARM, artificial rupture of membranes; C-section, caesarean section; SROM, spontaneous rupture of membranes with labor.

Table S2. Demographic and clinical characteristics of recruited pregnant women at term for the study with ELISA assay, qRT-PCR and Western blotting

	TNL (n=14)	TL (n=14)	*P* value
Maternal age (year)	32.57±0.56	31.43±0.54	0.15
Gestational age at delivery (week)	38.99±0.12	39.10±0.18	0.61
Delivery mode			
Vaginal (%)	0	100%	N/A
C-section (%)	100%	0	N/A
Membrane rupture			
SPOM (%)	0	100%	N/A
ARM (%)	100%	0	N/A
Gravidity median (range)	2 (1-5)	2 (1-3)	0.32
Parity median (range)	1 (1-2)	1 (1-2)	0.19
Fetal gender (male/female)	7/7	6/8	0.68
Birth weight (g)	3449.08±67.23	3277.14±99.04	0.16

TNL, term not in labor; TL, term in labor; ARM, artificial rupture of membranes; C-section, caesarean section; SROM, spontaneous rupture of membranes with labor.

Table S3. Demographic and clinical characteristics of recruited pregnant women at preterm for the study with ELISA assay, qRT-PCR and Western blotting

	PNL (n=10)	PL (n=10)	*P* value
Maternal age (year)	33.40±0.73	32.50±0.60	0.33
Gestational age at delivery (week)	34.50±0.34	33.46±0.59	0.12
Delivery mode			
Vaginal (%)	0	100%	N/A
C-section (%)	100%	0	N/A
Membrane rupture			
SPOM (%)	0	100%	N/A
ARM (%)	100%	0	N/A
Gravidity median (range)	1.5 (1-5)	1.5 (1-4)	0.46
Parity median (range)	1 (1-2)	1 (1-2)	0.55
Fetal gender (male/female)	6/4	5/5	0.68
Birth weight (g)	2241.50±112.77	2174.00±106.45	0.65

PNL, preterm not in labor; PL, preterm in labor; ARM, artificial rupture of membranes; C-section, caesarean section; SROM, spontaneous rupture of membranes with labor.

Table S4. Primer sequences used in qRT-PCR

Gene	Forward Primer (5’-3’)	Reverse primer (5’-3’)
ALOX15	GAAATTAACGTCCGGGCCA	CGATTCCTTCCACATACCGAT
ALOX15B	CTTCATAGCCACCCTCCCAC	AGGGGCCTTTGGTCTCCA
PTGS2	TGTGCAACACTTGAGTGGCT	ACTTTCTGTACTGCGGGTG
GAPDH	CCCCTCTGCTGATGCCCCCA	TGACCTTGGCCAGGGGTGCT
RELA	GGAAGGAACGCTGTCAGAGG	GGGTACTCCATCAGCATGGG
PTGES	GAAGAAGGCCTTTGCCAACC	GACGAAGCCCAGGAAAAGGA

Table S5 Abundance of AA metabolites in human amnion in TL and TNL

Metabolite Name	PGF2a	PGE2	15S-HETE	TXB2	AA	PGD2	6-keto-PG Fα	LTD4	12S-HETE	LTB4
QC RSD	0.048733	0.013481	0.060842	0.039727	0.039786	0.03144	0.048077	0.041643	0.024492	0.024859
TNL-1	3208.2	275828.3	26760.8	302073.1	2444641	23363.85	91293.12	81122.62	350623.5	9315.6
TNL-2	4241.408	285445.5	17681.93	325212.4	1654068	12738.95	114935	104481.7	60129.26	21104.83
TNL-3	1616.075	41973.95	6819.95	59605.33	2100336	3255.825	20177.96	16470.55	45234.96	6558.675
TNL-4	3897.65	118399.6	8744.8	119501.6	1738647	11930.35	60522.09	56436.24	79929.72	1942.077
TNL-5	5098.76	170286.4	17491.5	120497.7	2099995	24885.15	199624.7	145368.7	70697.66	17967.45
TNL-6	7010.55	206942.5	37934.33	495160.6	3867486	45211	338470.1	296231.6	124119.9	25215.45
TNL-7	3350.775	356640.1	55765.11	669099.7	2697273	20915.15	204948.1	214842	1589172	19376.93
TNL-8	4740.548	93377.69	20153.6	332280	2715887	51110.98	128834.6	113365.2	174981.3	44826.55
TL-1	10907.75	379342.6	43710.61	110183.1	1430383	6972.108	166760.5	169341.4	363282.8	11169.38
TL-2	5418	176982.7	13307.83	123107.1	950194.2	14616.08	45397.83	27904.05	65135.06	6178.625

TL-3	14353.88	706548	56716.11	219785.7	1487863	14093.4	120792.2	139654.5	697117.6	45443.7
TL-4	11003.48	514347.3	33156.78	142870	1499605	14640.28	41832.73	47543.53	93863.1	4277.825
TL-5	14733.85	535882.3	30375.23	119119.9	1628866	33294.74	39549.98	31185.73	55288.86	37748.43
TL-6	11549.38	163079.5	52200.31	98775.05	1450955	7704.861	81541.47	70085.14	65417.86	2768.958
TL-7	19840.45	1688620	162524.6	181252.6	2835172	21311.38	165149.4	219863.5	406932.9	14891.29
TL-8	26322.87	687092	80498.62	144792.5	2528907	19062.33	81543.07	75056.67	26501.74	5441.084
TL-9	5323.225	270825.8	35936.6	61745.28	2450729	5846.65	71790.54	60905.59	68414.19	11393.21
TL-10	14971.83	681600	50248.31	132027.5	1149180	16522.06	122356.4	104153.5	154476	15661.25
QC-1	11454.53	542115.4	42259.95	218126.6	2094846	21587.69	120410.3	107720.7	215671.2	17254
QC-2	11121.95	533041.1	47680.01	200034.7	2189673	22248.38	126549.5	111407.8	216183.9	17206.85
QC-3	11169.18	524979.5	48301.31	201651.7	1988103	20606.85	119261.7	109314.8	220639.9	16351.33
QC-4	10218.38	530183.8	44828.98	208448.3	2118126	21426.54	132203.6	118278.8	227304.9	16803.58
